# Scientific Items

**Published:** 1883-06-20

**Authors:** 


### SCIENTIFIC ITEMS.

**The Afflictions of the Spectroscope.**—A science, like a child, grows quickest in the first few years of its existence; and it is therefore not astonishing that, though twenty years only have elapsed since Spectrum Analysis first entered the world, we are able to speak to-day of a modern spectroscopy, with higher and more ambitious aims, striving to obtain results which shall surpass in importance any of those achieved by the old spectroscopy, to the astonishment of the scientific world.

A few years ago the spectroscope was a chemical instrument. It was the sole object of the spectroscopist, to find out the nature of a body by the examination of the light which that body sends out when it is hot. The interest which the new discovery created in scientific and unscientific circles was due to the apparent victory over space which is implied. No matter whether a body is placed in our laboratory or a thousand miles away—at the distance of the sun or of the farthest star—as long as it is luminous and sufficiently hot, it gives us a safe and certain indication of the elements it is composed of.

To-day, we are no longer satisfied to know the chemical nature of the sun and stars; we want to know their temperature, the pressure on their surface; we want to know whether they are moving away from us or toward us; and, still further, we want to find out, if possible, what changes in their physical and chemical properties the elements with which we are acquainted have undergone under the influence of the altered conditions which must exist in the celestial bodies. Every sun-spot, every solar prominence, is a study in which the unknown quantities include not only the physical conditions of the solar surface, but also the possibly changed properties, under these conditions, of our terrestrial elements. The spectroscope is rapidly becoming our thermometer and pressure gauge; it has become a physical instrument.

The application of the spectroscope to the investigation of the nature of celestial bodies has always had a great fascination to the scientific man as well as to the amateur; for in stars and nebulæ one may hope to read the past and future of our own solar system. But it is not of this application that I wish to speak to-day.

As there is no other instrument which can touch the conditions of the most distant bodies of our universe, bodies so large that their size surpasses our imagination, so is there no other instrument which equals it in the information it can yield on the minute particles at the other end of the scale, particles which in their turn are so small that we can form no conception of their size or number. The range of the spectroscope includes both stars and atoms, and it is about these latter that I wish to speak.—DR. ARTHUR SCHUSTER, *in Popular Scicuce Monthly.*

**Transit of Venus.**—We referred last month to the great astronomical event above named, and to its probable importance to the cause of science. We expressed our doubts about its ever benefitting the world commercially to the value of saving a single mariner's life, by adding to the accuracy of nautical investigations. In fact, the comparison of many different observations, at widely separate stations, have given very unsatisfactory results, making a difference of fully two minutes in the variation of the contact records when reduced to Washington time. This is a serious disappointment to science, to say the least, as it leaves more confusion than ever before as to the sun's actual distance from the earth. This, however, may possibly be partially, if not wholly, obviated by a comparison of the numerous photographs which were successfully taken at nearly all the stations where clear weather prevailed. Surely such exhibits, with the absolute time of their production, can scarcely fail to reconcile the unaccountable discrepancies in the contact records. But whatever the accuracy attained or attainable, we doubt if the result will ever justify the millions of dollars outlay to achieve it; especially while so many vastly more practical questions of science are ignored as not worthy of a thousandth part of the expenditure.—*Microcosm.*

**A Man** living at Bowling Green, Kentucky, who having attained the age of 29 years, and showing signs of dementation from a blow on the head in childhood, was placed under medical treatment, when by the trepanning process a depression in the skull was corrected and two pieces of bone were removed. As soon as the patient recovered from the effects of the chloroform which had been administered to him he was found to be perfectly rational, but his mind was utterly oblivious to every occurrence transpiring from the time he received the blow, though during the interval he had been married and had accumulated considerable property. The case is considered one of the most remarkable ever presented.—*Advocate.*

**It is reported** in the *Nature* that a discovery has been made near Andernach, on the Rhine. Remains of prehistoric animals have been found in a pumice-stone pit; and Prof. Schaaffhausen, of Bonn, has investigated the spot closely. A lava stream underlying the pumice-stone was laid bare, showing a width of only two metres. The crevices between the blocks of lava were filled with pumice-stone generally from the depth of one-half to one metre. Below this, however, there was pure loam and clay; and in this were found numerous animal bones, apparently broken by man, as well as many stone implements. It is supposed that there was a settlement there, of which the food remains fell into the crevices before the whole was covered with pumice-stone.—*Ibid.*

**A Swede** named Ditmer, has discovered and patented a process for solidifying kerosene. It is claimed that by it candles can be manufactured from kerosene so cheaply that the liquid article will be driven from our markets in Europe.—*Ibid.*

